# A Smartphone-Based sEMG Signal Analysis System for Human Action Recognition

**DOI:** 10.3390/bios13080805

**Published:** 2023-08-11

**Authors:** Shixin Yu, Hang Zhan, Xingwang Lian, Sze Shin Low, Yifei Xu, Jiangyong Li, Yan Zhang, Xiaojun Sun, Jingjing Liu

**Affiliations:** 1 College of Automation Engineering, Northeast Electric Power University, Jilin 132012, China; 2202100652@neepu.edu.cn (S.Y.); 201900461@neepu.edu.cn (H.Z.); 2202000549@neepu.edu.cn (X.L.); 2202000603@neepu.edu.cn (Y.X.); 2202100671@neepu.edu.cn (J.L.); 2202200607@neepu.edu.cn (Y.Z.); 2202100699@neepu.edu.cn (X.S.); 2 Research Centre of Life Science and HealthCare, China Beacons Institute, University of Nottingham Ningbo China, 199 Taikang East Road, Ningbo 315100, China; szeshin.low@gmail.com

**Keywords:** sEMG, HAR, deep learning, smartphone, rehabilitation

## Abstract

In lower-limb rehabilitation, human action recognition (HAR) technology can be introduced to analyze the surface electromyography (sEMG) signal generated by movements, which can provide an objective and accurate evaluation of the patient’s action. To balance the long cycle required for rehabilitation and the inconvenient factors brought by wearing sEMG devices, a portable sEMG signal acquisition device was developed that can be used under daily scenarios. Additionally, a mobile application was developed to meet the demand for real-time monitoring and analysis of sEMG signals. This application can monitor data in real time and has functions such as plotting, filtering, storage, and action capture and recognition. To build the dataset required for the recognition model, six lower-limb motions were developed for rehabilitation (kick, toe off, heel off, toe off and heel up, step back and kick, and full gait). The sEMG segment and action label were combined for training a convolutional neural network (CNN) to achieve high-precision recognition performance for human lower-limb actions (with a maximum accuracy of 97.96% and recognition accuracy for all actions reaching over 97%). The results show that the smartphone-based sEMG analysis system proposed in this paper can provide reliable information for the clinical evaluation of lower-limb rehabilitation.

## 1. Introduction

During the rehabilitation of lower limbs, rehabilitation therapists provide immediate feedback and guidance to patients after observing them perform designated actions [1]. However, the real-time guidance of therapists is often influenced by subjective factors such as feelings, experiences, and judgments, which bring some errors. Additionally, the rehabilitation service provided by therapists is limited by time and resources, making it impossible to monitor and guide multiple patients simultaneously or for a long time. It may delay rehabilitation processes [2,3]. Fortunately, the introduction of sensing information has provided strong support for implementing this task, allowing rehabilitation therapists to accurately evaluate patients’ actions based on quantitative experimental data. In this way, the rehabilitation efficiency was improved significantly [3,4,5,6]. Currently, research on action recognition based on human physiological electrical signals is receiving widespread attention [7,8,9,10]. Among them, surface electromyography (sEMG) signals are widely used due to noninvasive collection and the ability to reflect the activity of limb muscles [11,12,13,14].

Nowadays, recognition systems based on sEMG signals mostly rely on signal acquisition devices in laboratories or medical institutions. Moreover, they are limited to offline analysis of data on the PC end. There is a lack of real-time monitoring and timely feedback on patients’ muscle function and gait posture during rehabilitation [15,16,17]. This undoubtedly reduces the convenience of implementing lower-limb rehabilitation plans and the flexibility of adjusting plans, which may indirectly increase rehabilitation costs for patients. Therefore, there is a need for a portable miniaturized sEMG collection and analysis system that can collect data conveniently and offer real-time analysis of collected sEMG data for patients and rehabilitation teams to make timely adjustments to abnormal situations [18,19]. The emergence of mobile human–machine data interaction interfaces caters to this demand. They help in completing action recognition tasks in a more efficient and simple manner, thereby helping patients recover as soon as possible.

As one of the most widely used mobile devices, smartphones have powerful multicore processors, internet connection, and efficient operating systems, which can serve as a convenient platform for point-of-care-testing (POCT). Therefore, in this work, we introduced smartphones to develop a portable sEMG sensor monitoring and analysis system for lower-limb rehabilitation. The system consists of two main components: (1) a self-made distributed small sEMG device paired with a Bluetooth low-energy (BLE) module specifically designed for continuous signal monitoring, which can collect and transmit data of subjects during normal walking; (2) an Android-based application (app), with multiple functions for recording, displaying, and analyzing sEMG signals in real time, which can also output action recognition results, allowing users or caregivers to easily access information through graphics.

To build the dataset required for this recognition model, an experiment was designed to collect the sEMG signal segment of different lower-limb actions. To cater to the demands of lower-limb rehabilitation, six actions (kick, toe off, heel off, toe off and heel up, step back and kick, and full gait) were designed to form the experimental action set. These six actions transition from simple single-muscle movements to coordinated movements of multiple muscle groups, ultimately achieving continuous walking. The gradient motion design from simple to difficult can help rehabilitation personnel gradually recover lower-limb muscle function and gait coordination and can thus be used to achieve more comprehensive and effective rehabilitation training.

A convolutional neural network (CNN) was introduced as a representative of deep learning models. Its ability to automatically extract features caters to the needs of complex high-dimensional data processing, which has shown excellent performance in multiple human action recognition (HAR) studies [20,21,22]. Even in the face of a large amount of unlabeled data, CNNs can still achieve accurate classification. Additionally, CNNs have outstanding transferability, which can be transferred to new activities without labelling [23,24]. Also, a specific method for capturing muscle contraction starting point was proposed to achieve precise segmentation of sEMG signal caused by single action, which bridges the differences in individual actions, and pursue the common features of the same action sEMG initial response point, thereby improving the quality of the dataset, and further optimizing the training results of the CNN. Then, an easy-to-use human–machine interaction interface was built into the app, where the CNN can be invoked to enable the smartphone to display action recognition results online.

The results indicate that the system can provide rich information directly related to body activities, which are references for improving lower-limb rehabilitation training. The feasibility of the monitoring system was verified through experimental testing.

## 2. Materials and Methods

### 2.1. sEMG Signal Acquisition Device

In this work, a highly reliable distributed wearable sEMG device was developed, which consists of one master controller and three slave computers. The master controller c, an Internet of Things chip ESP32 module (Espressif, Shenzhen, China) that integrates Bluetooth and a high-performance dual-core processor, responsible for receiving and preprocessing sEMG signals, which is connected to the terminal directly, while each slave computer consists of three parts: a power supply and signal transmission board, an sEMG signal preprocessing board, and a commercial disposable electrode.

The power supply and signal transmission board mainly include ESP32, TP5400 (lithium battery charge and discharge management chip), and their peripheral circuits. Among them, the power management module enables the device to work independently and stably. In the wireless communication module, ESP-NOW, a low-power wireless communication protocol from Espressif was introduced. In the design of the sEMG signal preprocessing board, to unify the intensity differences of different muscles’ sEMG signal, which would make it hard to observe signals at the same level, an amplifier (with three operational amplifiers (AD8236ARMZ, AD8646ARMZ, AD8648ARUZ) as the core, combined with peripheral circuits) was configured in the sEMG signal preprocessing board to adjust the output gain of the sEMG signal so that the response curve thresholds of different channels were basically on the same magnitude. The electrode, the lower part, can be attached to the subject’s skin through adhesive foam and flexible conductive paste, while the upper part is connected to the metal buckle to complete signal transmission.

**Figure 1:**
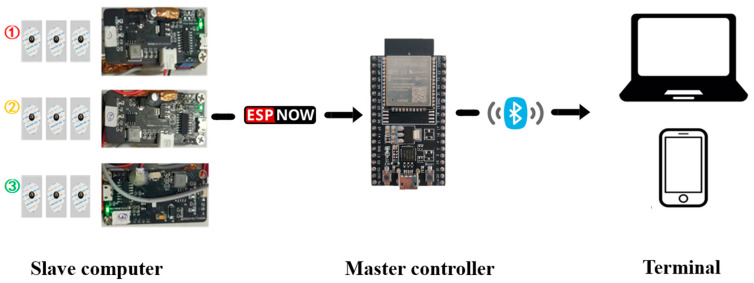
Portable electromyographic device and its communication method.

When the target muscle contracts, nerve impulses in the muscle fibers cause the muscle cells to release an electrical charge, generating a weak electrical signal. The sensing system, with a differential mode input impedance of 110 GΩ, a common-mode rejection ratio of 110 dB, and a sampling rate of 500 Hz, can sense the weak potential changes on the muscle surface by means of the attached Ag/AgCl electrodes. The quality of the sEMG signals can be further improved using a differential circuit and a signal amplifier. Then, the signal transmission board transmits the signal to the master controller through the ESP-NOW communication protocol. After receiving the data from each slave computer, the master controller integrates signals and sends them to the terminal (PC or smartphone) through a Bluetooth module for display and analysis (as shown in Figure 1).

### 2.2. Data Acquisition

#### 2.2.1. Selection of Muscle Groups

**Figure 2:**
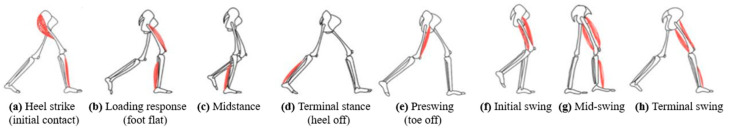
Illustration of a complete gait cycle [25].

Taking walking posture during limb rehabilitation as reference, multiple muscle groups in the legs during normal walking were selected. As shown in Figure 2, a complete gait can be divided into eight stages (a–h), in which stage (a) is mainly the contraction of the gluteus medius and tibialis anterior muscles; stage (b) is mainly the contraction of the quadriceps femoris muscle; stage (c) and (d) are mainly the contraction of the muscles behind the lower leg; stage (e) is mainly the contraction of the rectus femoris, plantaris flexor, and toe flexion muscles; stage (f) is mainly the contraction of the iliopsoas muscles; and stage (g) and (h) are mainly the contraction of the quadriceps femoris muscle, biceps femoris muscle, and tibialis anterior muscles. In order to obtain strong and highly correlated sEMG signals, it is required to select muscle groups as large as possible, which should be located on the surface, and easy-to-place electrodes. Three muscle groups, rectus femoris, anterior tibialis, and gastrocnemius muscle, were selected from the above muscle groups.

#### 2.2.2. Selection of Collection Points

**Figure 3:**
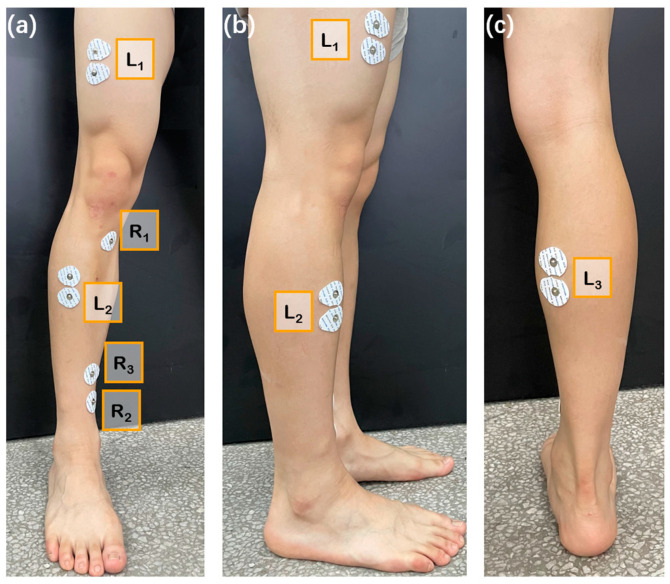
Specific positions of action electrodes and reference electrodes attached to three muscle groups: (**a**) front view; (**b**) side view; (**c**) back view.

In this paper, three electrodes were required to measure a single muscle area. Two electrodes were placed on the selected muscle, and the other electrode, as reference, was placed where the influence by muscle activity is small. Based on the analysis of human muscle tissue, the knee, tibia, and ankle regions were selected as the positions for reference electrodes, the locations of the other two electrodes attached to the surface of three muscle groups, wherein L_1–3_ successively represents the locations of the action electrodes attached to the rectus femoris, anterior tibialis, and gastrocnemius muscles; R_1–3_ sequentially represents the reference electrode positions of the three muscle groups (as shown in Figure 3 (presented from triple perspectives of front, side, and back)).

For different muscle groups, the direction of attachment of the sEMG electrodes (the line between the two action electrodes) should be parallel to the muscle fibers, whereas the position of attachment of the electrodes (the position of the electrode center) should be located in a line connecting the beginning and the end of the muscle, whose precise position can be determined by the longitudinal and transversal directions: longitudinally, the electrodes should be placed at the midway point between the most distal motor endplate area and the distal tendon; transversally, the electrodes should be placed on surfaces far away from the edges of the muscles so that the geometrical distances from the target muscle to the other muscles can be maximized.

#### 2.2.3. Experiments

Twelve healthy college student subjects (4 females and 8 males, from Jilin, China) were invited to participate in the experiments. All subjects’ leg muscle strength levels were 3 and above. Their age range is 23–28 years; their height range is 175–182 cm, and weight range is 52–98 kg. The subjects, right-handed and with no known neuromuscular or sensory disorder, were previously informed of the specifics of the experiment and signed an informed consent form. The experiment is divided into two parts. Experiment 1 collected the sEMG signals of a single action, while experiment 2 collected a test dataset closer to the real control scenario. 

**Figure 4:**
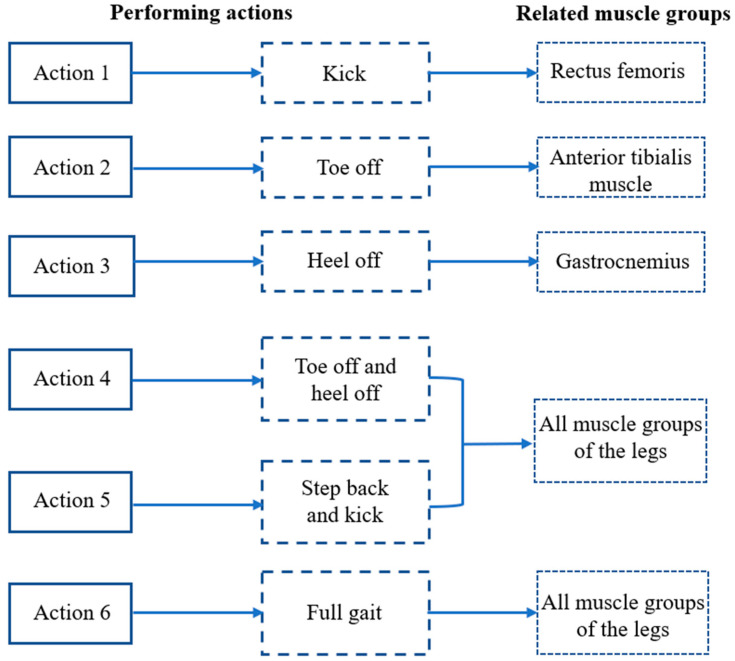
Execution requirements for 6 actions.

In Experiment 1, 8 subjects (A1–A8) were selected for 4 experiments each, with electrodes attached to the surface of the designated muscles at least 6 h apart. During this period, the subjects were required not to engage in vigorous exercise to ensure that the muscles returned to the same initial state. In a single experimental cycle, each subject was required to perform action 1–6 (action requirements are shown in Figure 4) 120 times, and each action needed to be completed within the execution cycle (5 s). After that, the subject was allowed to rest for 15 min.

In Experiment 2, 6 subjects (A1, A2, B1–B4) were selected to perform random actions 100 times. At each moment, the subject performs a single random action based on the reported information.

### 2.3. Smartphone-Based sEMG Signal Analysis System

**Figure 5:**
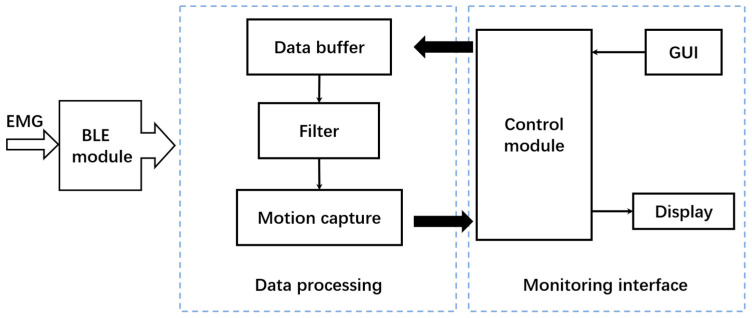
Software processing framework for the EMG data.

Considering the convenience and widespread acceptance of smartphones, an app for sEMG signal monitoring and analysis was developed. The software processing framework is shown in Figure 5. After the electrodes are attached to the target muscle of the subject, the collection device and smartphone-based app work separately. EMG data are obtained from the slave computers; then, the real-time monitoring interface runs, and the slave computers send the data to the master controller through ESP-NOW. After that, the Bluetooth module inside the master controller starts and sends the integrated 3-channel data to the data preprocessing module. Then, the data buffer completes the storage of real-time data, and the control module of the monitoring interface completes the display of sEMG time-varying data. In the data processing part, the filter completes the filtering of peak noise. Meanwhile, the users can activate the action capture function through the button on the GUI interface, which could capture a single action signal segment through feature extraction and storage the data before sending it to the control module. The control module further analyzes the data segment, displays the waveform of the single action data segment and the action recognition result.

### 2.4. Data Processing

In this work, data processing involves the following steps: first, the sEMG signals obtained in experiment are divided into a set of single action signal segments by using muscle contraction starting point capture method; second, the data are filtered to remove high-frequency harmonic components; finally, all samples are integrated and the outliers are removed from the sEMG data.

#### 2.4.1. sEMG Data Partition Based on Muscle Contraction Starting Point Capture Method

**Figure 6:**
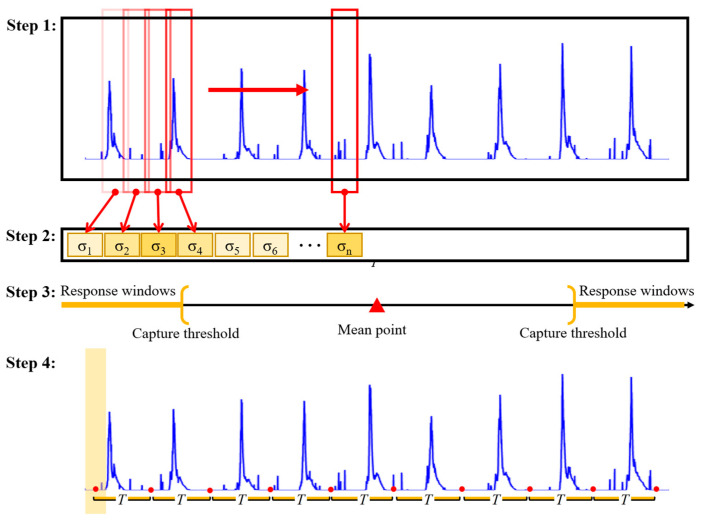
Main steps of muscle contraction starting point capture method.

A statistics-based method was proposed to capture the start point of muscle contraction from EMG data. The proposal of this method draws on the 3σ criterion of normal distribution, and a similar approach has been applied to analyze human physiological signals [26]. According to the 3σ principle of normal distribution, the likelihood of an event occurring in the range [μ−σ,μ+σ] is 68%, the likelihood of an event occurring in the range [μ−2σ,μ+2σ] is 95%, and the likelihood of an event occurring in the range [μ−3σ,μ+3σ] is 99.7%, which encompasses virtually all possibilities. In this work, combining probabilistic theory and mathematical statistics, the dispersion of the muscle in the resting state is regarded as the mean μ of a normal distribution, the mean of the standard deviation of all the windowed sEMG data is taken as σ, and the data exceeding 3σ are the anomalous datapoints (the onset nodes of the muscle’s stretching and contraction). In the actual data processing process, given the individual variability, we then proposed an adaptive capture factor θ to optimize the capture range, which resulted in the final capture principle. The start point is used as the reference point for extracting segments from sEMG data. The main steps are as follows (as shown in Figure 6).

Step 1: The standard deviation of processed sEMG signals for each segment is calculated through a sliding window. The standard deviation calculation method is shown in Equation (1). Compared to resting state, muscle contraction is more significant during exercise, which can be observed in sEMG signals. Therefore, the standard deviation of each window can effectively describe the degree of variation in sEMG signals.

(1) $$\sigma =\sqrt{\frac{\sum_{i=1}^{l}{\left(x_{i}-\bar{x}\right)}^{2}}{l}}$$

where $x_{i}$ is the value of each datapoint in the window, $\bar{x}$ is the mean value of all data in the window, and l is the number of datapoints in the window. 

Step 2: The data within all windows are calculated to form a standard deviation sequence, where the standard deviation of each subsegment shows the degree of its dispersion on the complete sEMG signal. This provides an important basis for capturing the initial moment caused by muscle contraction.

Step 3: Based on the dispersion index and the principle of 3σ in normal distribution, the probability of events occurring in [μ−3σ,μ+3σ] is 99.7%, so the values exceeding 3σ are considered outliers. Herein, the mean value of the normal distribution μ is the dispersion of the sEMG signal segment in the resting state. When the subject executes the action, the fluctuation data generated by the muscle show different dispersion compared with the data in the resting state. By setting the capture threshold, the start time of muscle contraction is captured. The two key parameters, relative dispersion $\sigma_{d}$ and capture factor θ are introduced, where relative dispersion provides the basic boundary for describing window differences and prominence, and its calculation method is shown in Equation (2).

(2) $$\sigma_{d}=\sqrt{\frac{\sum_{i=1}^{n}{\left(\sigma_{i}-\bar{\sigma }\right)}^{2}}{n}}$$

where $\sigma_{i}$ is the standard deviation of each window, $\bar{\sigma }$ is the mean value of the standard deviations of all windows, and n is the number of all windows. 

After obtaining these two key parameters, the response window, the window where the muscle contraction starts, is finally determined through Equation (3). The center point of this window is selected as the initial moment of muscle contraction.

(3) $$Response\;Windows=\{W_{k}|\sigma_{k}>\bar{\sigma }+\theta \cdot \sigma_{d},\sigma_{k}\in S\}$$

where $W_{k}$ is the response window, k is the standard deviation of the kth window, S is the set of standard deviation sequences, and $\bar{\sigma }$ is the mean value of the standard deviation sequence.

Step 4: After determining the initial moment of muscle contraction, the specified length of the segment is continuously intercepted based on the execution cycle of different actions, thereby completing the segmentation task of all sEMG data segments.

#### 2.4.2. Filtering

High-frequency noise interferes with signals during signal acquisition; it is random and has no correlation with the response signal. Moreover, the high-frequency noise greatly reduces the recognition accuracy. Therefore, a lowpass filter with a cutoff frequency of 50 Hz is used to attenuate high-frequency noise, and a median filter is used to remove outliers, reduce artifact interference, and further improve the signal-to-noise ratio of the data. In this way, relatively smooth signals are obtained. These applied filters are implemented by using customized scripts in MATLAB (MathWorks, Natick, MA, USA).

### 2.5. CNN

The precise classification of 6 actions is the core of the entire sEMG analysis system. In this work, a CNN is used for feature extraction to classify the sEMG signals of given actions. Instead of relying on the hand-designed features of traditional machine learning algorithms, a CNN adopts a multilayered neural network structure to autonomously carry out feature extraction, learning, and hierarchical representation, which enables the model to adapt to different data situations and is more robust at the theoretical level [27,28]. Additionally, introducing activation functions makes the model flexible and adaptable when dealing with complex data in nonideal states (electrode offset, sweat interference), thus making the whole system better applied in practice.

#### 2.5.1. Network Structure

TensorFlow, the deep learning library, was introduced. The CNN model was built by using the computing environment provided by Spyder. After data preprocessing, the experimental data should be processed to make the input size conform to of the model. The 3-channel specified length sEMG data segments are used as inputs, their corresponding action labels are used as outputs, and sparse categorical cross-entropy is introduced as a loss function to calculate the cross-entropy loss between the predicted and true labels.

**Figure 7:**
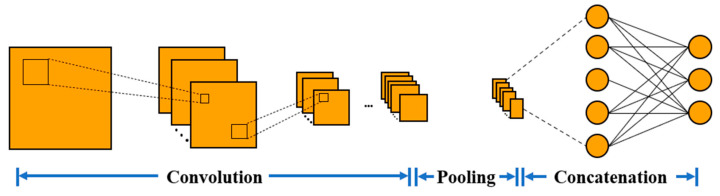
CNN network structure.

The CNN consists of four convolutional layers and three fully connected layers (as shown in Figure 7). The first convolutional layer extract local time-domain features from the sliced sEMG data. Then, higher-level features are extracted through the second, third, and fourth layers of convolution. Meanwhile, the batch normalization and ReLU activation functions are used to increase the robustness of the model. After that, the pooling layer is used to reduce the dimension of each feature map of the input vector. Then, those features are combined by the last three full connection layers. The dense layer multiplies the feature tensor and the weight and adds the offset term. The dropout layer randomly zeros some of the divine elements to prevent overfitting. Finally, the dense layer uses the Softmax activation function to convert the output value into a probability distribution and output classification results for 6 different actions. It is worth noting that the pooling layer used here is a global average pooling of the output of the convolution layer. It has no trainable parameters, so as to reduce the number of parameters in the full connection layer, improves the generalization ability of the model, and further reduces the risk of overfitting of the model.

#### 2.5.2. Hyperparameter

In the training process, the batch size was 10, the learning rate (LR) was 0.001, and the epoch was 45. An Adam optimizer was used to optimize convolutional kernel parameters and update weights. The evaluation metric was accuracy. During training, the model saved the optimal weights, the weights with the best performance on the validation set through a callback function. If the performance of the model on the validation set does not improve after 12 iterations, the training is be stopped, and the optimal weights are restored.

## 3. Results

### 3.1. Original sEMG Data

Taking the raw sEMG training dataset of subject A1 as an example, the sEMG response curves for each action in multiple response cycles are shown in Figures S1–S6. The abscissa in the figure represents time, where the negative value represents the time before the stopping recording timepoint. The ordinate represents the voltage difference obtained from two action electrodes, which can be used to represent the intensity of muscle contraction. It can be observed that during the execution of the actions, the amplitude of the electromyographic signal may suddenly increase and form a peak. These spikes are usually short in duration and may exhibit sharp peaks, which are random and have no correlation with the response signal of the set action.

### 3.2. Preprocessing Results

After data partition from the original sEMG training data of subject A1 by using the muscle contraction starting point capture method, the single-action sEMG data segment was fully extracted. Then, the interference of peak noise was effectively eliminated through filtering.

**Figure 8:**
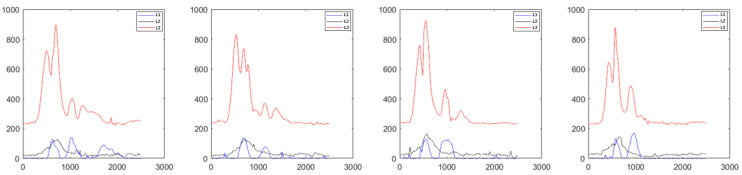
Sample data filtering during A1 subject executing action 1 (red: response curve of sEMG at rectus femoris muscle; black: response curve of sEMG at tibialis anterior; blue: response curve of sEMG at gastrocnemius muscle).

As shown in Figure 8 (taking the first four samples of A1 subject performing action 1 as an example), a relatively smooth sample signal was obtained, and good consistency was shown in multiple single-action data segments.

### 3.3. Sample Selection

The original sEMG data contain some samples that deviate significantly from the standard sample signal. When these nonstandard samples are fed into the model for training, the CNN recognition model has a larger recognition range for the subject’s actions, which can lead to misidentification of nonstandard actions. Furthermore, due to the fact that standardized actions have been set, it can affect the effectiveness of rehabilitation. Therefore, it is necessary to eliminate nonstandard samples.

**Figure 9:**
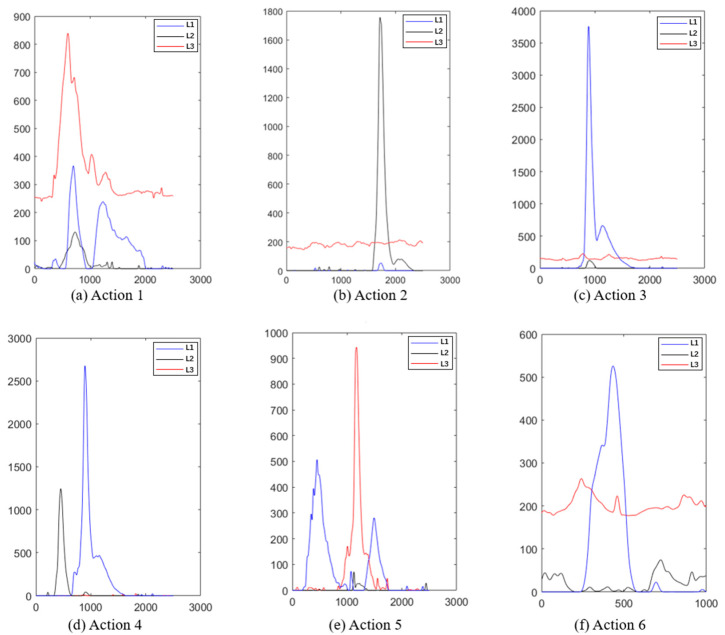
Filtered response curve of sEMG signals during subject A1 executing each action (red: response curve of sEMG at rectus femoris muscle; black: response curve of sEMG at tibialis anterior; blue: response curve of sEMG at gastrocnemius muscle).

As shown in Figure 9, the response intensity recorded from each muscle during different action execution are different. However, the response trend shows significant consistency within each segment. Taking actions 2 (toe off) and 4 (toe off and heel off) as examples (as shown in Figure 9b,d), during the execution of action 2, channel 2, recording the intensity of rectus femoris contraction, there is a very short duration peak, corresponding to the brief process of rectus femoris contraction causing the toe off. During the execution of action 4, channel 2 and channel 3 (tibialis anterior muscle) showed peaks in turn, and the amplitude of these two channels decreased rapidly. Channel 1 (gastrocnemius muscle) was almost stable near the baseline because of its minor correlation with this action. In addition, we also added the sEMG response curves of another subject (A2) in the support file (as shown in Figure S7), whose signal trend showed good agreement with that of subject A1. However, a slight difference in the signal amplitude of the sEMG response between subjects A2 and A1 during performing actions 2 and 5 can be found, which was caused by individual muscle strength differences. Typically, in the case of a person of approximate age, individuals with greater muscular capacity will contract their corresponding muscles more strongly when performing the same movement, resulting in a greater EMG signal amplitude [29].

The stable values (corresponding to resting potential), peaks (corresponding to muscle contraction ability), and durations (corresponding to the speed of action execution) of the response waveform within the normal range are beneficial variables for the recognition model. However, waveforms with significant differences in the number of peaks, duration, and peak values in the sample are abnormal samples that need to be removed. The abnormal samples mainly include abnormal response peak size (much larger than the normal response peak, abnormal response peak (with additional response peaks), and no response peak (without response during the execution cycle). The waveform diagrams are shown in Figure S7a–c, which have been removed from the dataset.

### 3.4. Performance Evaluation

**Figure 10:**
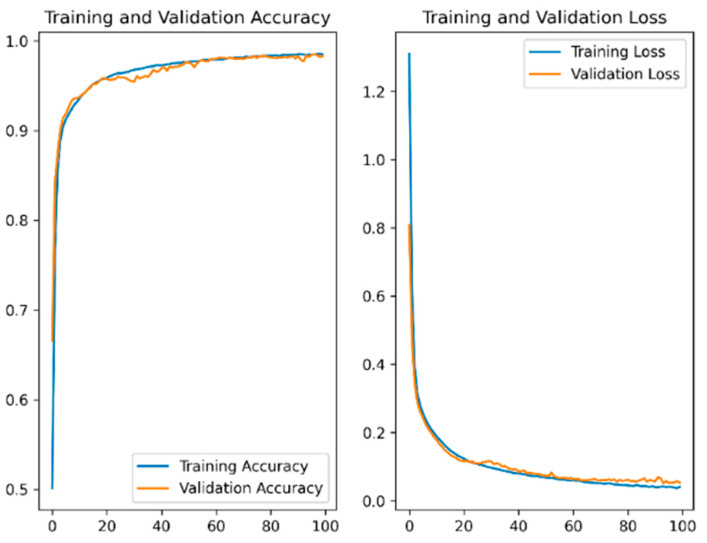
Training results of CNN sEMG recognition model.

The sEMG data collected through experiments were used to train the CNN recognition model. The training curve converged, as shown in Figure 10. The highest accuracy rates during the training phase were 97.96%.

**Figure 11:**
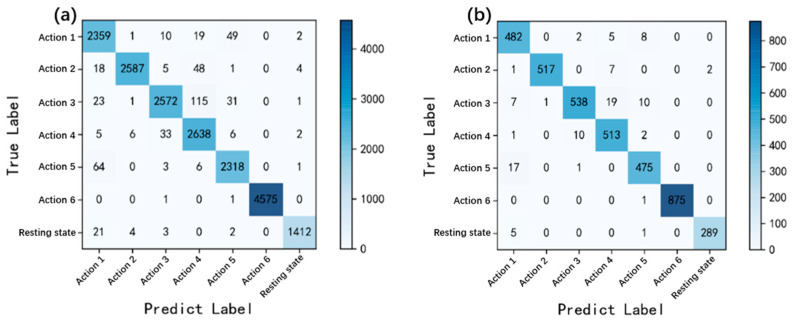
Confusion matrix: (**a**) training set; (**b**) test set.

After the training of the CNN recognition model, the confusion matrix was introduced to evaluate the classification effect of the model. Two groups of data samples were selected to evaluate the confusion matrix. One is the training set samples, whose results are shown in Figure 11a. The results can be used to measure the applicability of the CNN recognition model to the classification of subjects who provide CNN training samples. The second is the test set samples from the second part of the sEMG signal acquisition experiment, as shown in Figure 11b. The samples of subjects B1 to B4 in experiment 2 did not participate in the model training process; the recognition result can used to represent the recognition accuracy of the model for the action of users who have never used the sEMG recognition system.

According to the confusion matrix in the Figure 11, the secondary indicators of the model include accuracy rate, accuracy, precision, recall, and F1 Score. The analysis results are shown in the Table 1. According to Table 1, the classification accuracy of the model in both the training and testing sets has reached over 97%, indicating excellent classification performance of the CNN recognition model.

## 4. Mobile Interface

In this work, Qt, an application development framework was introduced. A software platform was developed for analyzing sEMG signals by introducing multithreading technology [30,31]. Qt provides a rich toolset and class libraries, enabling developers to develop an app in C++ or other programming languages (such as Python). Its cross-platform characteristics facilitate the deployment of sEMG signal analysis systems on multiple platforms.

**Figure 12:**
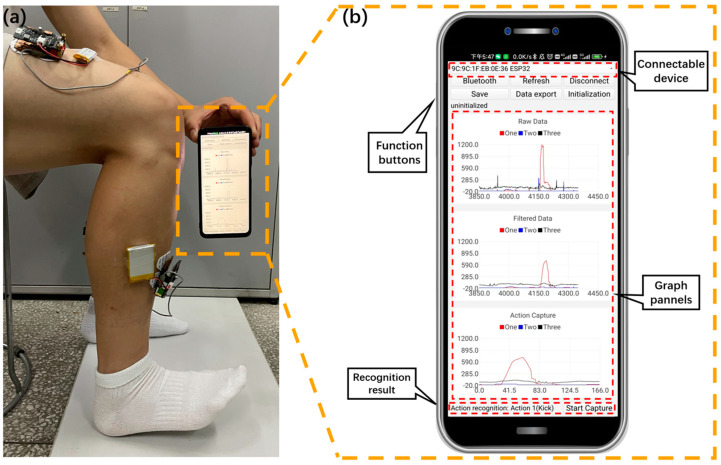
Monitoring the execution of action 2 (toe off) through the sEMG signal analysis app: (**a**) actual measurement image; (**b**) mobile interface.

The established CNN recognition model is further integrated into the mobile interface. In addition, the app allows users to observe the activity intensity and feature changes of lower-limb muscles during action execution, as shown in Figure 12a. This app has convenient interactive capabilities and is easily accepted by users. Before using the app, we briefly explained to the subjects the basic functions of the app and how to use it, and throughout the experimental phase, no subjects were troubled by the use of the app. As shown in Figure 12b, the application interface consists of three graphical panels and seven buttons, with a total of five main functions, including Bluetooth communication, filtering, data export, action capture, and action recognition.

### 4.1. Bluetooth Communication

The Bluetooth function in the app interface includes activating Bluetooth communication, refreshing connectable devices, and connecting to specified devices. As shown in Figure 12, when the users click the Bluetooth button, the corresponding event processing function is triggered. In the event processing function, call the API function to start the Bluetooth communication function and initialize the Bluetooth adapter. The “Refresh” button is used to refresh the list of all Bluetooth devices that can be connected within the communication range, scan the surrounding Bluetooth devices, obtain the list of connected devices, update the device list or dropdown menu in the application interface, and display the available devices for selection. The “Connect” button is used to connect the application to the specified Bluetooth device. When the users select the device to be connected and clicks the “Connect” button, a connection is established with the selected device. After successful connection, update the application interface to reflect the connection status and take subsequent actions.

### 4.2. Filtering

The median filtering function is introduced into the app, which accepts the original sEMG data as input and returns the data after median filtering. After receiving the original sEMG data, it is transferred to the median filter function for processing. The median filter function filters each datapoint and replaces it with the median value of the data in its neighborhood. This can effectively eliminate outlier and spikes. Use the drawing module of Qt to display the filtered sEMG data curve in the app interface. The filtered data were used to update the plot to display the smoothed curve after median filtering.

### 4.3. Data Export

The “Save” button can be used to record the time that the button was clicked based on a timestamp, which is recorded as a node timepoint. At this point, the recording variable is set to true and data recording begins. During the data acquisition process, the status of the recording variable is monitored. If the recording variable is true, it indicates that data are being recorded. Store each collected datapoint in memory or a temporary array until the “Data export” button is clicked again. When the users click this button, set the recording variable to false to terminate recording data. Afterwards, convert the previously recorded data to .CSV format and save it to a file. According to requirements, saved CSV files can be sent out through network communication protocols (such as TCP/IP) or other appropriate methods.

### 4.4. Action Capture

The app obtains original sEMG data from devices through Bluetooth modules. These data are usually collected continuously in the form of a timeseries. In order to capture EMG signal segments that execute a single action, the app needs to divide the entire collected dataset into multiple segments. Using the “Start capture” button, when the triggering conditions are met, the application starts extracting the current sEMG signal segment, intercepting a specific time window or index range from the collected data to obtain the signal segment for executing a single action. In the application interface, the processed signal segments are displayed for visual observation by users. Additionally, the signal segment can be saved to a file for future offline analysis.

### 4.5. Action Recognition

The action capture module processes and encapsulates the captured single-action segment data and sends the data to the Python script server by using the Bluetooth communication protocol. A server-side script written based on Python can be used to receive sEMG data sent by the master controller. The socket of Python is used to connect the server and app. After receiving the data, they are passed to CNN recognition model for action recognition. After receiving the sEMG data sent by the master controller on the server, they are input into the trained CNN model for action recognition and the results are obtained. Then, the result of action recognition is sent back to the app through a Python script. As shown in Figure 12a, the subject is performing action 2 (toe off), and after the mobile app captures the information, the model sends the recognition results back to the app for display with low latency (as shown in Figure 12b).

In summary, this app has practical functions such as monitoring visualization, detailed recording, transmission, and processing of sEMG data. In addition, the collected data can be used to further analyze more available information, such as lower-limb action recognition results, standardized action execution prompts, and so forth. Based on this, a smartphone-based sEMG signal analysis system was successfully established by combining self-made collection devices.

## 5. Conclusions

In this work, a wearable sEMG sensing module was developed, which utilizes a distributed multichannel communication system and wireless communication module to achieve direct connection between the detection device and the mobile phone, enabling the system to achieve real-time monitoring of sEMG data in rehabilitation scenarios. At the same time, a specific sEMG response timepoint capture method was proposed to adapt to the differences in muscle responses among different subjects, providing an effective dataset for CNN-based lower-limb action recognition models, thereby achieving feature extraction of complex sEMG signals and completing the classification task of six different leg postures. The results showed that the recognition accuracy during the training period was 97%. Subsequently, by using a CNN, the smartphone was equipped with action recognition function based on real-time sEMG signals, which further provided substantial assistance for portable electromyographic signal analysis research and necessary data support for patient behavior evaluation during the rehabilitation process. Although the existing structural volume of the sEMG instrument cannot meet the needs of sEMG signal detection for shallow small muscle groups, the volume needs to be further reduced to expand its application scenarios.

In conclusion, this work carried out experimental flow design, software, and hardware development in the context of rehabilitation research, and initially validated the idea of real-time action recognition based on sEMG signals on smartphones. However, in order to indeed apply this system to the disabled population in the future, it is necessary to increase the experimental samples of disabled people to improve the portability of the CNN model. However, the ability of real-time collection and excellent performance in analyzing sEMG data of this system gives us the confidence to believe that the continuous monitoring system of physiological electrical signals based on smartphones will become an important development trend in HAR research. Furthermore, it will be used as a powerful booster to promote the wider application of related research in the healthcare field.

## Figures and Tables

**Table 1:** CNN Recognition Model Evaluation.

	Index	Action 1	Action 2	Action 3	Action 4	Action 5	Action 6	Resting State
Training set	Recall	0.9668	0.9715	0.9377	0.9807	0.9691	0.9996	0.9792
	Precision	0.9474	0.9954	0.9791	0.9335	0.9626	1.0000	0.9930
	F1 score	0.9570	0.9833	0.9579	0.9565	0.9658	0.9998	0.9860
	Accuracy	-	-	-	0.9743	-	-	-
Test set	Recall	0.9698	0.9810	0.9260	0.9753	0.9635	0.9989	0.9797
	Precision	0.9396	0.9866	0.9764	0.9430	0.9557	1.0000	0.9931
	F1 score	0.9545	0.9838	0.9505	0.9589	0.9596	0.9994	0.9863
	Accuracy	-	-	-	0.9721	-	-	-

## Data Availability

Research data are not shared.

## Supplementary Materials

The following supporting information can be downloaded at: https://www.mdpi.com/article/10.3390/bios13080805/s1, Figure S1. The original sEMG time-varying curve of A1 subject executing action 1 (red: response curve of sEMG at rectus femoris muscle; black: response curve of sEMG at tibialis anterior; blue: response curve of sEMG at gastrocnemius muscle). Figure S2. The original sEMG time-varying curve of A1 subject executing action 2 (red: response curve of sEMG at rectus femoris muscle; black: response curve of sEMG at tibialis anterior; blue: response curve of sEMG at gastrocnemius muscle). Figure S3. The original sEMG time-varying curve of A1 subject executing action 3 (red: response curve of sEMG at rectus femoris muscle; black: response curve of sEMG at tibialis anterior; blue: response curve of sEMG at gastrocnemius muscle). Figure S4. The original sEMG time-varying curve of A1 subject executing action 4 (red: response curve of sEMG at rectus femoris muscle; black: response curve of sEMG at tibialis anterior; blue: response curve of sEMG at gastrocnemius muscle). Figure S5. The original sEMG time-varying curve of A1 subject executing action 5 (red: response curve of sEMG at rectus femoris muscle; black: response curve of sEMG at tibialis anterior; blue: response curve of sEMG at gastrocnemius muscle). Figure S6. The original sEMG time-varying curve of A1 subject executing action 6 (red: response curve of sEMG at rectus femoris muscle; black: response curve of sEMG at tibialis anterior; blue: response curve of sEMG at gastrocnemius muscle). Figure S7. Filtered response curve of sEMG signals of subject A2 executing each action (red: response curve of sEMG at rectus femoris muscle; black: response curve of sEMG at tibialis anterior; blue: response curve of sEMG at gastrocnemius muscle). Figure S8. Abnormal sample waveform.
