# Supplementary material for: A Smartphone-Based sEMG Signal Analysis System for Human Action Recognition
Source: Biosensors (Basel). 2023 Aug 11;13(8):805. doi: 10.3390/bios13080805 (PMC10452551; doi:10.3390/bios13080805)
Supplement: Supplementary file 1 [file biosensors-13-00805-s001.zip › biosensors-2477323-supplementary.pdf]

biosensors

Supplementary Material

A Smartphone-Based sEMG Signal Analysis System for Human Action Recognition

Shixin Yu [1], Hang Zhan [1], Xingwang Lian [1], Sze Shin Low [2], Yifei Xu [1], Jiangyong Li [1], Yan Zhang [1], Xiaojun Sun [1] and Jingjing Liu [1,*]

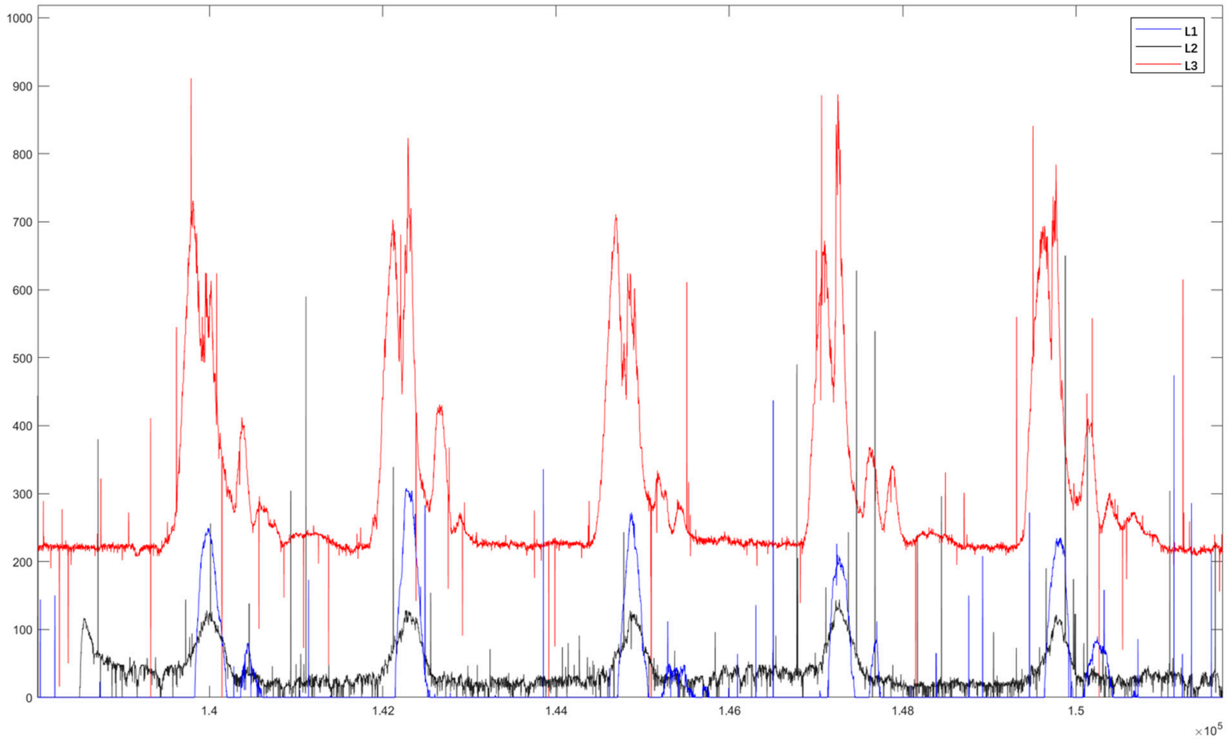

Figure S1. The original sEMG time-varying curve of A1 subject executing action 1 (red: response curve of sEMG at rectus femoris muscle; black: response curve of sEMG at tibialis anterior; blue: response curve of sEMG at gastrocnemius muscle).

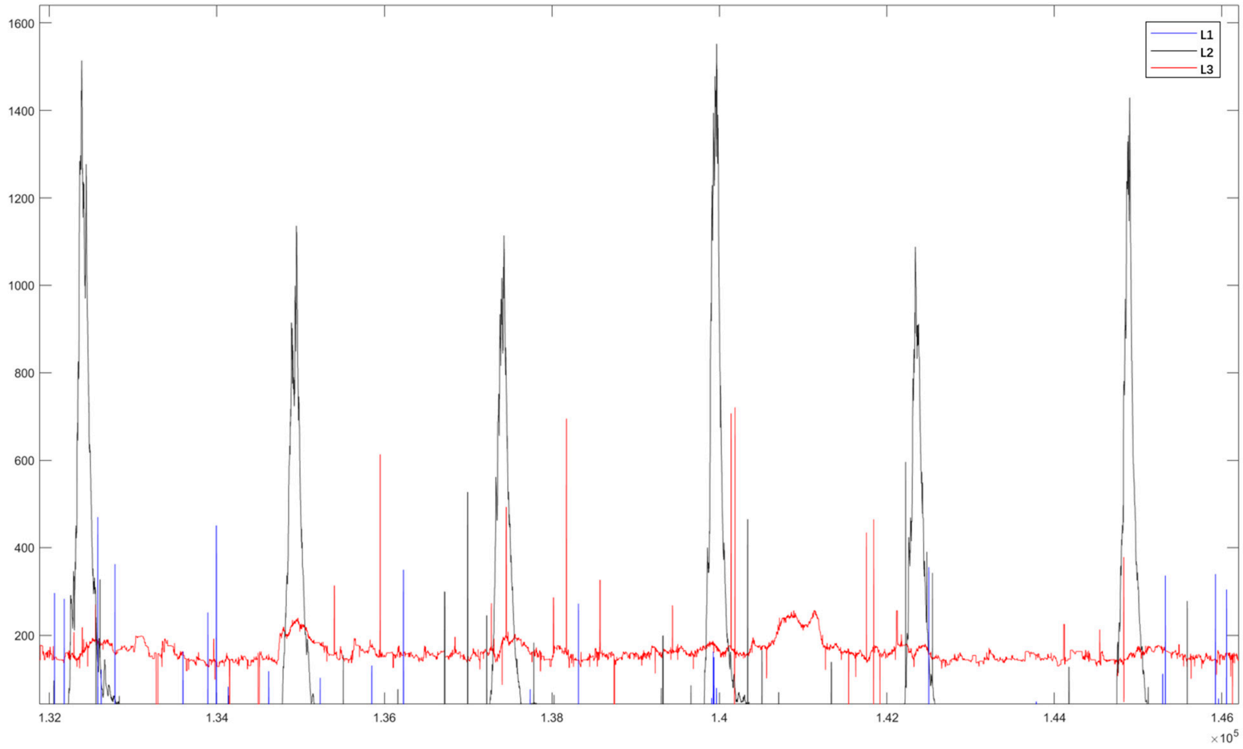

Figure S2. The original sEMG time-varying curve of A1 subject executing action 2 (red: response curve of sEMG at rectus femoris muscle; black: response curve of sEMG at tibialis anterior; blue: response curve of sEMG at gastrocnemius muscle).

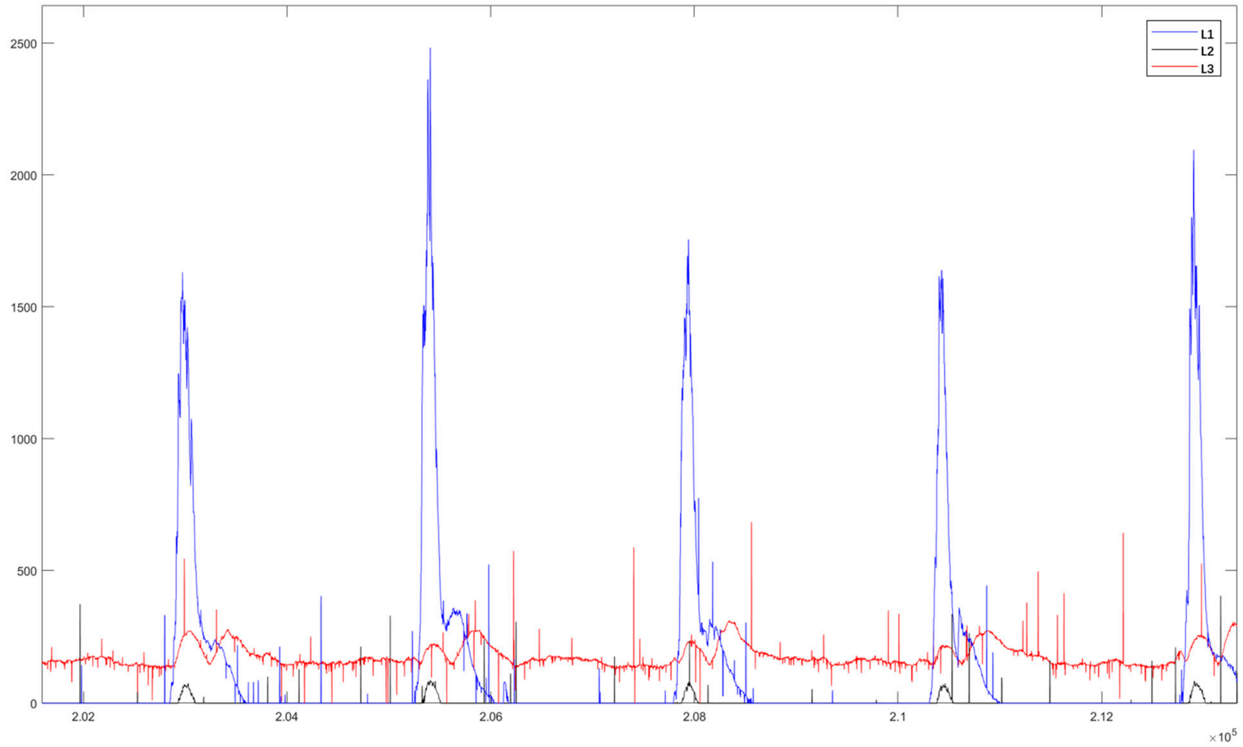

Figure S3. The original sEMG time-varying curve of A1 subject executing action 3 (red: response curve of sEMG at rectus femoris muscle; black: response curve of sEMG at tibialis anterior; blue: response curve of sEMG at gastrocnemius muscle).

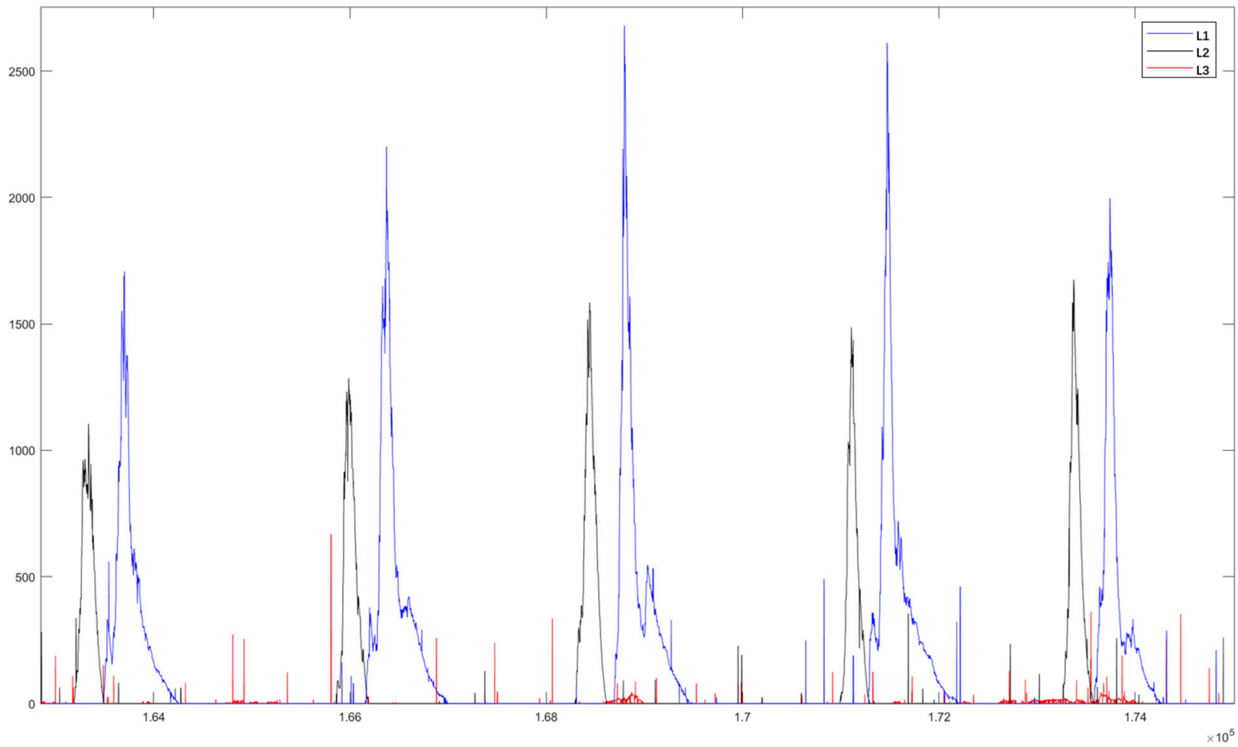

Figure S4. The original sEMG time-varying curve of A1 subject executing action 4 (red: response curve of sEMG at rectus femoris muscle; black: response curve of sEMG at tibialis anterior; blue: response curve of sEMG at gastrocnemius muscle).

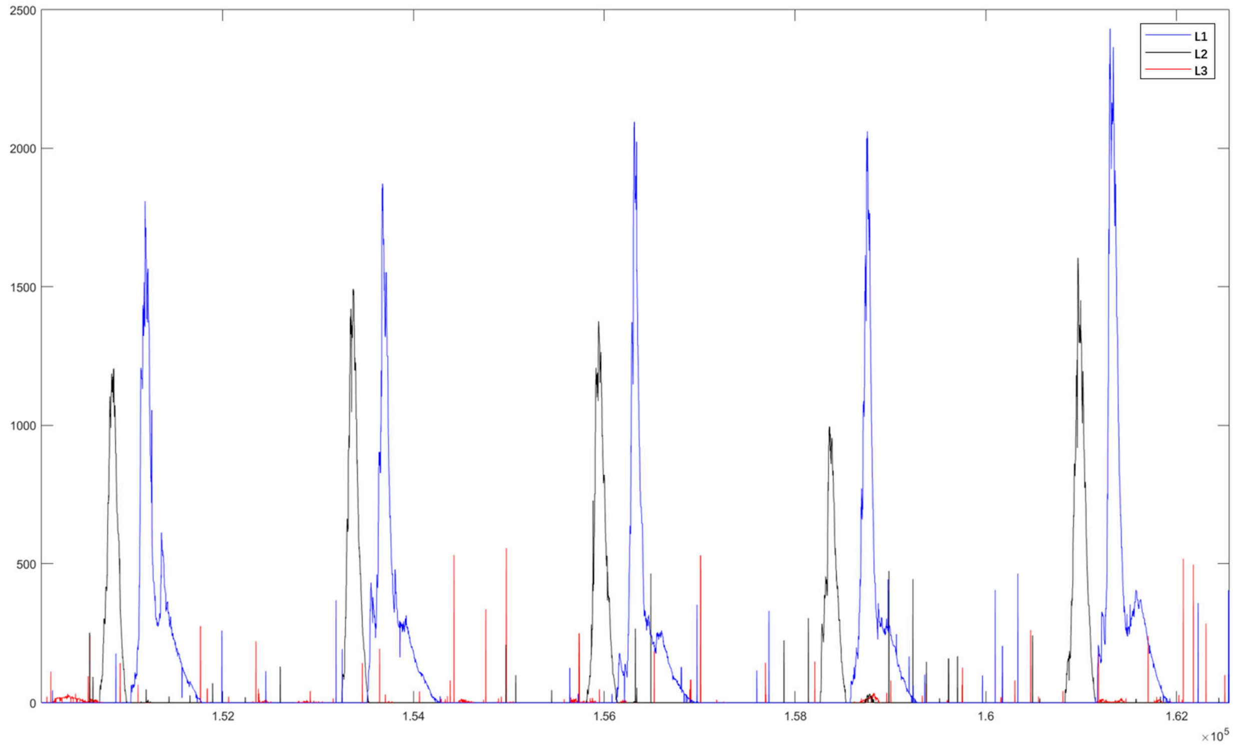

Figure S5. The original sEMG time-varying curve of A1 subject executing action 5 (red: response curve of sEMG at rectus femoris muscle; black: response curve of sEMG at tibialis anterior; blue: response curve of sEMG at gastrocnemius muscle).

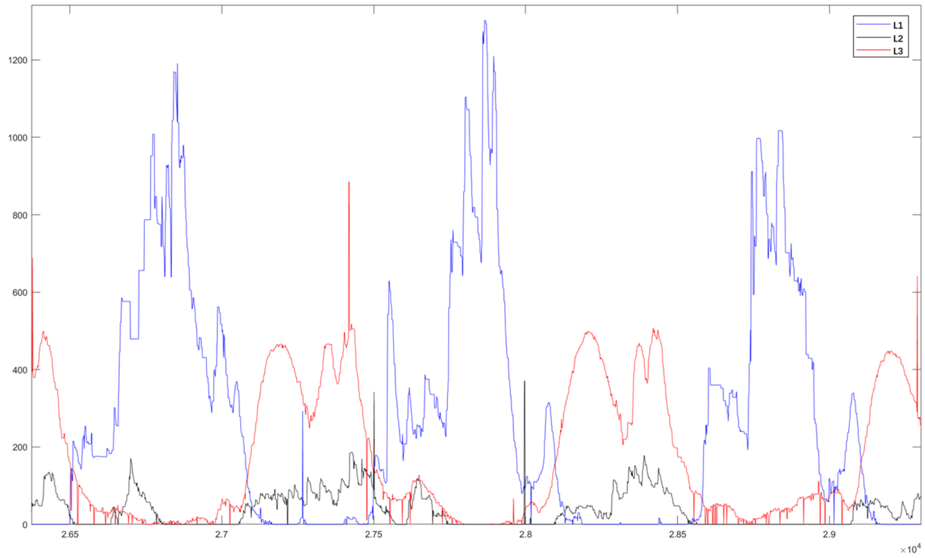

Figure S6. The original sEMG time-varying curve of A1 subject executing action 6 (red: response curve of sEMG at rectus femoris muscle; black: response curve of sEMG at tibialis anterior; blue: response curve of sEMG at gastrocnemius muscle).

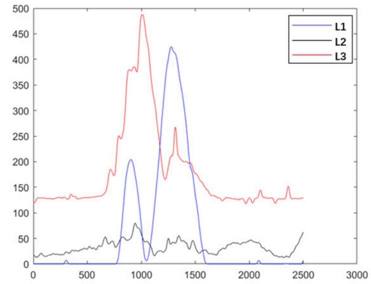

(a) Action 1

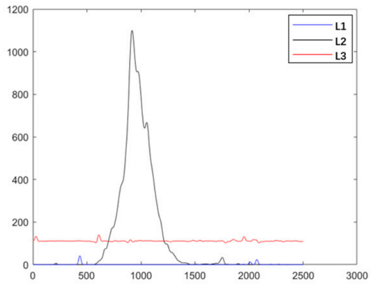

(b) Action 2

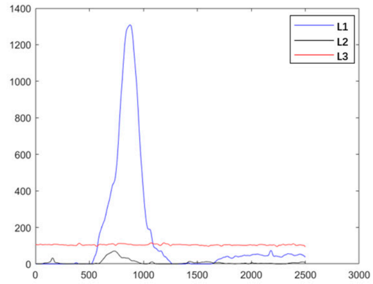

(c) Action 3

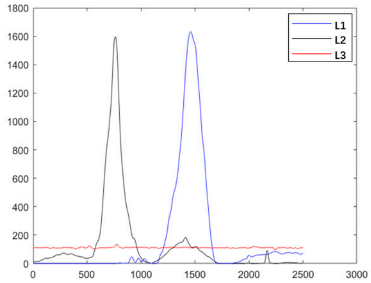

(d) Action 4

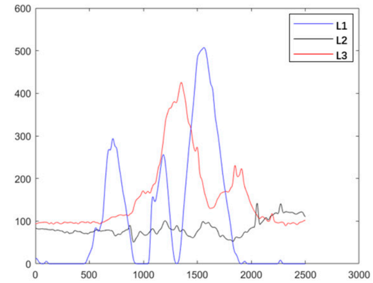

(e) Action 5

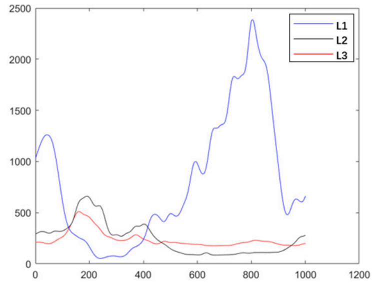

(f) Action 6

Figure S7. Filtered response curve of sEMG signals of subject A2 executing each action (red: re-sponse curve of sEMG at rectus femoris muscle; black: response curve of sEMG at tibialis anterior; blue: re-sponse curve of sEMG at gastrocnemius muscle).

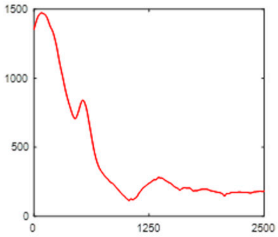

(a) Peak anomaly

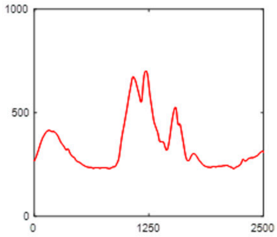

(b) Peak number anomaly

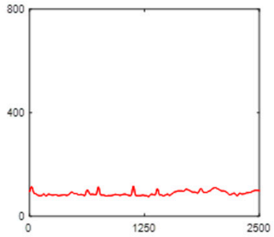

(c) No peaks

Figure S8. Abnormal samples waveform.
